# Rational Design of Multifunctional Ferulic Acid Derivatives Aimed for Alzheimer’s and Parkinson’s Diseases

**DOI:** 10.3390/antiox12061256

**Published:** 2023-06-11

**Authors:** Eduardo Gabriel Guzmán-López, Miguel Reina, Luis Felipe Hernández-Ayala, Annia Galano

**Affiliations:** 1 Departamento de Química, Universidad Autónoma Metropolitana-Iztapalapa, Av. Ferrocarril San Rafael Atlixco 186, Col. Leyes de Reforma 1A Sección, Alcaldía Iztapalapa, Mexico City 09310, Mexicohdz.ayala@quimica.unam.mx (L.F.H.-A.); 2 Departamento de Química Inorgánica y Nuclear, Facultad de Química, Universidad Nacional Autónoma de México, Mexico City 04510, Mexico

**Keywords:** rational design, antioxidants, electron transfer, hydrogen transfer, neuroprotection, AChE, COMT, MAOB

## Abstract

Ferulic acid has numerous beneficial effects on human health, which are frequently attributed to its antioxidant behavior. In this report, many of them are reviewed, and 185 new ferulic acid derivatives are computationally designed using the CADMA-Chem protocol. Consequently, their chemical space was sampled and evaluated. To that purpose, selection and elimination scores were used, which are built from a set of descriptors accounting for ADME properties, toxicity, and synthetic accessibility. After the first screening, 12 derivatives were selected and further investigated. Their potential role as antioxidants was predicted from reactivity indexes directly related to the formal hydrogen atom transfer and the single electron transfer mechanisms. The best performing molecules were identified by comparisons with the parent molecule and two references: Trolox and α-tocopherol. Their potential as polygenic neuroprotectors was investigated through the interactions with enzymes directly related to the etiologies of Parkinson’s and Alzheimer’s diseases. These enzymes are acetylcholinesterase, catechol-O-methyltransferase, and monoamine oxidase B. Based on the obtained results, the most promising candidates (FA-26, FA-118, and FA-138) are proposed as multifunctional antioxidants with potential neuroprotective effects. The findings derived from this investigation are encouraging and might promote further investigations on these molecules.

## 1. Introduction

Oxidative stress (OS) is a harmful multifaceted phenomenon, often referred to as the “chemical silent killer” since no evident symptoms are associated with it. To this day, there is no available test to detect it. Thus, its damaging effects can evolve without any advice to the affected person. Currently, OS represents a major concern linked to the onset and development of hundreds of illnesses. Among the available strategies to lessen OS risks to human health, chemical protection by antioxidant molecules is one of the most effective and studied approaches. Antioxidants can be seen as sacrificial compounds that prevent oxidants from reaching biomolecules. Antioxidants are produced endogenously by the human body and can be acquired through the intake of food and dietary supplements.

**Scheme 1:**
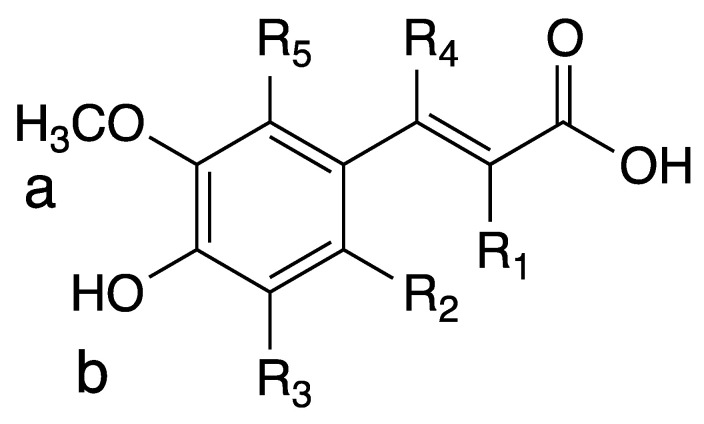
Ferulic acid (FA, R_1_ = R_2_ = R_3_ = R_4_ = R_4_ = H) structure and site numbering used in this work.

Ferulic acid (4-hydroxy-3-methoxy cinnamic acid, FA, Scheme 1) is one of these valuable molecules. It is found in whole grains, grapes, parsley, rhubarb, spinach, cereal seeds, artichoke, and coffee, among many other natural sources [1]. It is a versatile molecule. There are numerous reports on its antioxidant activity [2,3,4,5,6,7,8,9,10,11,12,13,14] as well as on its anti-inflammatory [15,16], antibacterial [17,18,19,20], antiviral [21], anti-thrombotic [22,23], anti-ageing [24,25,26], and antitumoral effects [27,28,29,30,31,32,33,34,35,36,37,38,39,40,41]. It also acts as a cardio protector [42,43,44,45,46,47,48], neuroprotector [49,50,51,52,53,54,55,56,57,58,59], antihypertensive [60,61,62,63], antidepressant [51,64,65,66,67,68,69], hepatoprotector [70,71,72,73,74,75,76,77,78,79,80,81], and has beneficial effects on diabetes [82,83,84,85,86,87,88] and gentamicin-induced nephrotoxicity [89].

Thus, it is not surprising that many efforts have been devoted to the development of FA derivatives [21,27,29,90,91,92,93,94,95,96,97,98,99,100,101,102,103,104,105,106,107,108,109,110,111,112,113,114,115,116,117,118,119,120,121,122,123,124,125,126,127,128,129,130,131,132,133,134,135]. Some of their relevant structural modifications and properties are summarized in Table 1. Their bioactivities are diverse, including antioxidant, anticancer, anti-inflammatory, and neuroprotective effects. According to the gathered data, it becomes evident that the ferulic acid molecular framework is a promising choice for developing new molecules with health benefits.

In this work, a systematic and rational search for FA derivatives is presented. For that purpose, a computer-assisted protocol known as CADMA-Chem [136] was used. The goal of the search is to find candidates that behave as multifunctional antioxidants, which are currently recognized as promising candidates to deal with OS-related complex diseases. This kind of antioxidants can scavenge free radicals, chelate metals and inhibit OH production, repair oxidatively damaged biomolecules, and inhibit enzymes involved in the development of health disorders [136,137,138]. In particular, the derivatives designed in the present work are meant to be oral drugs that simultaneously act as neuroprotectors against Parkinson’s and Alzheimer’s diseases, as well as free radical scavengers. It seems worthwhile to emphasize the fact that they are novel structures and that such combined activity has not been previously reported. In pursuit of such a goal, the FA framework was modified through the inclusion of different functional groups at all R1 to R5 sites (Scheme 1). Absorption, distribution, metabolism and excretion (ADME) properties were evaluated, as well as toxicity and synthetic accessibility (SA). Antioxidant activity through electron and H donation was predicted. Polygenic protection was explored by the interaction with enzymes linked to the target diseases. Namely: acetylcholinesterase (AChE), monoamine oxidase type B (MAOB), and catechol-O-methyltransferase (COMT). The inhibition of the first one has been shown to help with Alzheimer’s [139,140,141], while the inhibition of the other two is beneficial for Parkinson’s [142,143,144,145,146,147,148,149]. The obtained results are encouraging and might promote further investigations on the molecules identified as the most promising candidates.

## 2. Computational Details

### 2.1. Molecular Properties

For all the designed ferulic acid derivatives (Table S1), physicochemical parameters related to absorption, distribution, metabolism and excretion (ADME) were evaluated (Table S2) with the Molinspiration Property Calculation Service [150] and RDKit software (RDKit: Open-source cheminformatics. https://www.rdkit.org, accessed on 25 May 2023) The computed parameters are employed to confirm if the designed derivatives satisfy the Lipinski’s, Ghose’s, and Veber’s rules [151,152,153]. Compounds violating more than one of Lipinski’s or Veber’s rules are assumed to have difficulties with bioavailability, while those violating Ghose’s may present absorption problems or low permeation. Viable medical drugs also need to fulfill other vital requirements, such as synthetic accessibility (SA) and safety. The SA of the designed compounds was determined with the SYLVIA-XT 1.4 program (Molecular Networks, Erlangen, Germany) [154,155]. It estimates a value between 1 and 10. The smaller the value, the easier it is to synthesize the compound. LD_50_ and Ames mutagenicity (M) were employed to assess the toxicity of FA and its derivatives. The Toxicity Estimation Software Tool (T.E.S.T.), version 4.1 [156], was employed for that purpose. Selection and elimination scores (Tables S2 and S3), expressed in terms of toxicity, manufacturability and ADME properties, were used for sampling the molecular space. A reference set of molecules, which have been used (to some extent) as neuroprotectors, was used for comparison purposes (Table S4). 

### 2.2. Reactivity Indexes

Gaussian 09 package of programs was employed for electronic structure calculations [157]. The M05-2X/6-311+G(d,p) level of theory was used for geometry optimizations and frequency calculations. The solvation model density (SMD) [158] was used for solvent effects, using water as a solvent. Local minima were identified by the absence of imaginary frequencies, and unrestricted calculations were used for open shell systems. M05-2X is a wide-spectrum functional with good performance for noncovalent interactions, thermochemistry and kinetics [159]. In addition, it has been recommended for modeling open-shell systems [160]. M05-2X functional has also been successfully used to estimate bond dissociation energies (BDE) and the free radical scavenging activity of diverse antioxidants [161,162,163,164,165].

The electron propagator theory (EPT) [166,167] was used to calculate ionization energies (IE) and electron affinities (EA). The partial third-order quasiparticle theory (P3)^112^ was chosen, within the EPT framework, because it produces lower mean errors than other approaches [168]. Pole strength (PS) values were checked to be larger than 0.80–0.85 (Table S5), which validates the obtained results [169]. 

For the estimation of BDEs, all sites likely to act as H-atom donors were considered, i.e., the -CH_3_ in the ether moiety of FA, and the phenolic OH (sites *a* and *b*, Scheme 1) and the new groups arising from functionalization of R1 to R5 sites. 

Acid constants, expressed as pKa, were calculated with the Marvin suite [170]. This property is of crucial importance for medical drugs since it governs the proportion of neutral species at a particular pH, and these are the species most likely to passively cross biological barriers. The reliability of Marvin estimations was validated. To that purpose, 137 pKa were estimated, which correspond to first and second deprotonations of phenols, amines, carboxylic acids, thiols, and compounds structurally close to ferulic acid. The molecules used for such a validation are those reported in references [171,172,173]. The mean unsigned error (MUE) obtained from comparisons with the corresponding experimental data was found to be 0.42 pKa units. The correlation between calculated and experimental pKa values is provided in Supplementary Figure S1 (slope = 1.01, intercept = 0.04, and R^2^ = 0.95). This seems to support the reliability of the pKa values estimated here with the Marvin software. 

### 2.3. Enzymatic Interactions 

The structures of COMT (PDB ID: 3S68), MAO-B (PDB ID: 2V5Z) and AChE (PDB ID: 4EY7) co-crystallized with recognized neuroprotector drugs, tolcapone, safinamide and donepezil, respectively, were obtained from the protein data bank [174,175,176]. AChE missing loop regions (256-PGGTGG-261 and 493-PKA-496) were fixed using the Modeller web service [177]. Water molecules and species without biological interest were removed with the Discovery Studio software [178]. Ionizable groups of protein were modelled considering the protonation state of lateral chains and charge for D, E, R, K and H amino acids at physiological pH. For ligands, atomic charges are estimated by NBO protocol as single-point calculations with DFT (M05-2X/6-311+G(d,p)) methodology. Docking simulations were carried out using AutoDock Vina software [179]. A gradient optimization algorithm was performed inside of the active site centered at x: −13.50, y: 37.69, z: 61.63 and grid size of 15 × 15 × 15 Å^3^ for COMT, x: 51.81, y: 156.34, z: 28.15 and grid size of 13 × 13 × 13 Å^3^ for MAO-B and x: −18.80, y: −43.83, z: 27.67 and grid size of 17 × 13 × 13 Å^3^ for AChE. Docking scores (ΔG_B_^W^) were reported for the best-docked pose and weighted according to the abundance (molar fraction) of the acid-base species at physiological pH. The best conformation was analyzed and drawn with Pymol 2.5.4 software [180]. 

The redocking RMSD values were 1.8, 1.6 and 2.8 Å, respectively, and redocking scores (7.65, 10.10 and 10.86 kcal/mol) were found for tolcapone (COMT), safinamide (MAO-B) and donepezil (AChE), respectively, which agrees with experimental findings. These results confirm the suitability of the docking methodology. Redocking conformations are obtained with Chimera software [181], and they can be found in Figure S2.

## 3. Results and Discussion

### 3.1. Derivatives and Properties

By inserting -OH, -NH_2_, -SH and -COOH groups in sites R_1_ to R_5_, 185 new FA derivatives were built (Table S1). Twenty of them with one functional group, one hundred and sixty with any possible combination of two functional groups, and five with three functional groups. The latter were constructed from the most promising bi-functionalized species. 

A selection score (S^S^) was computed. It is meant to identify the FA derivatives with the most likely drug-like behavior and corresponds to that included in the CADMA-Chem protocol [136,137,138,182,183,184]. The associated equations are provided in Table S6. The higher the value of S^S,^ the more likely the drug-like behavior. S^S^ takes into account eight ADME properties: water/octanol partition coefficient (logP), topological polar surface area (PSA), number of heavy atoms (^X^At), molecular weight (MW), number of H-bond acceptors (HB^A^), number of H-bond donors (HB^D^), rotatable bonds (RB), and molar refractivity (^M^R); two toxicity descriptors: median lethal dose for rats (LD_50_) and Ames’ mutagenicity (M); and the synthetic accessibility (SA).

**Figure 1:**
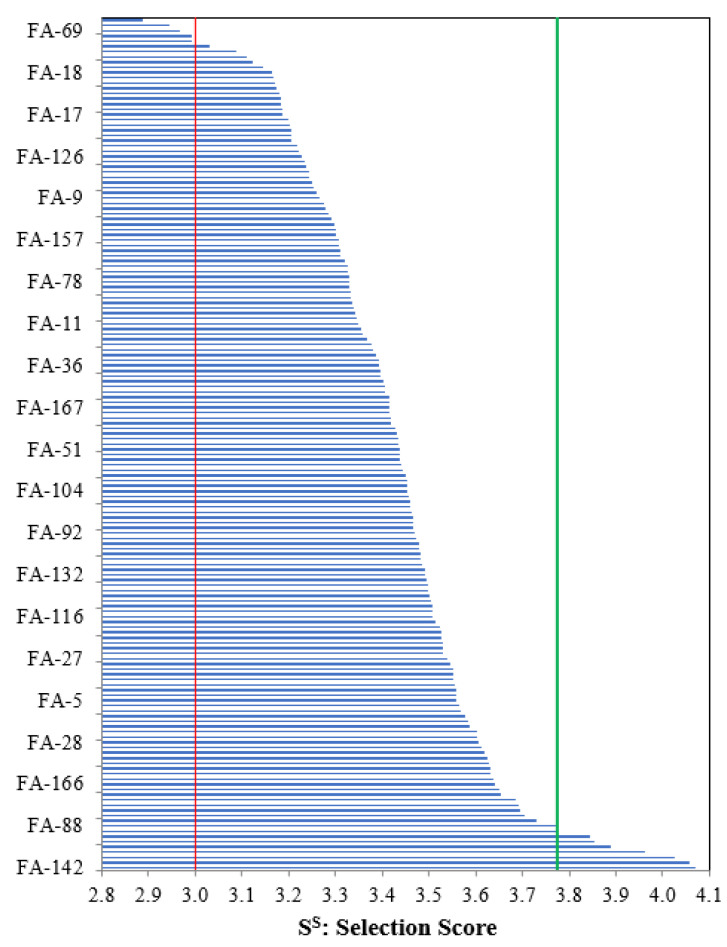
Selection score (S^S^) for the FA derivatives designed in this work. Vertical lines mark the arithmetic mean of the reference set (red) and the value for the parent molecule (FA, green).

**Scheme 2:**
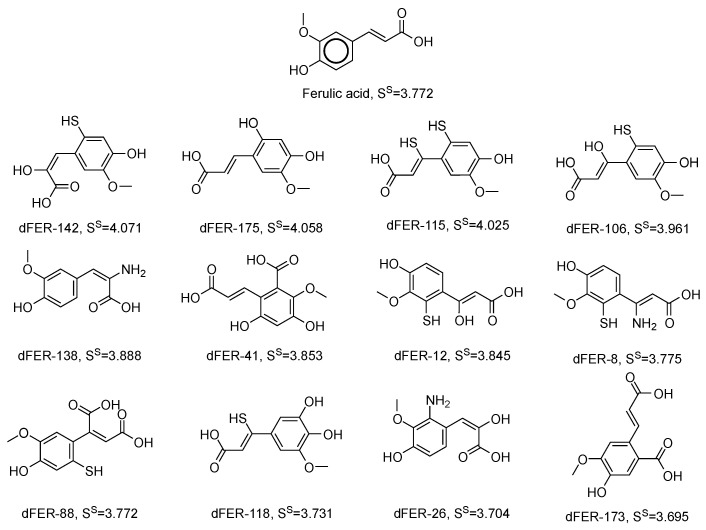
Structure and S^S^ values of FA and the derivatives selected for the next stage of the investigation.

The S^S^ for all the designed FA derivatives is presented in Figure 1. The parent molecule and the average S^S^ value for the reference set are included for comparison purposes. The individual values of all the FA derivatives are reported in Table S2, together with those of the above-mentioned descriptors. Higher values of S^S^ suggest better drug-like behavior, lower toxicity, and easier synthesis. The first screening was based on this score, and twelve FA derivatives were selected. However, before moving them forward to the next stage of the investigation (Scheme 2), a double-check analysis was performed using exclusion scores (S^E^), which allowes to verify if any of the selected molecules significantly deviate (in any of its properties) from the average value of the reference set.

Four exclusion scores were analyzed (S^E,ADME2^, S^E,ADME8^, S^E,ADMET^ and S^E,ADMETSA^). Their equations are provided in Table S7. S^E,ADME8^, S^E,ADMET^ and S^E,ADMETSA^ are extensions of the well-known S^E,ADME2^, based on two descriptors (logP and MW) [185,186]. S^E,ADME8^ uses the same kind of strategy as S^E,ADME2^, but includes six additional terms (PSA, XAt, HB^A^, HB^D^, RB, and ^M^R). S^E,ADMET^ and S^E,ADMETSA^ also include toxicity (LD_50_ and M) and synthetic accessibility (SA) descriptors. 

S^E,ADME2^ values were previously estimated to be between 1.2 and 1.5 for 1791, 152 and 35 oral drugs [185,186]. For the 12 selected FA derivatives, the average S^E,ADME2^ value was found to be 1.06, with individual values ranging from 0.56 to 1.42 (Table S4). The estimated average values for the other elimination scores were found to be: S^E,ADME8^ = 4.86 (ranging from 2.59 to 7.34), S^E,ADMET^ = 8.13 (ranging from 4.53 to 11.54), and S^E,ADMETSA^ = 9.59 (ranging from 5.50 to 12.52). It seems worthwhile mentioning that high values of the exclusion scores might result from either worse or better behavior than the average of the reference drugs. Thus, a detailed analysis is required to determine if any particular candidate should be removed from the selection or not.

**Figure 2:**
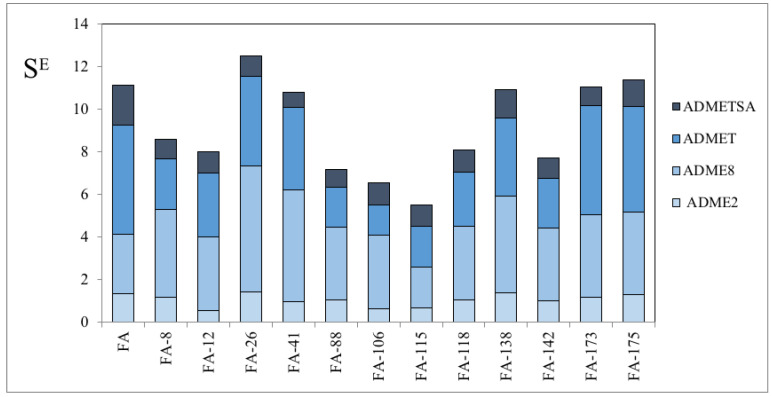
Elimination score (S^E^) for the most promising FA derivatives, according to S^S^. Columns are divided to show the influence of the new contributions included in each score, with respect to the previous one.

**Figure 3:**
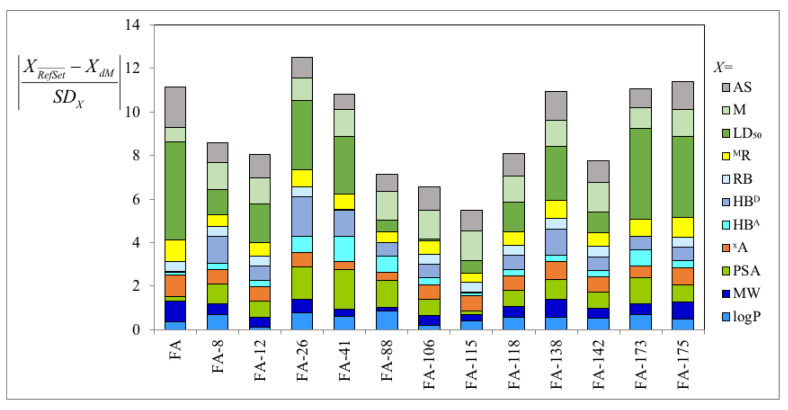
Individual contributions to the elimination score (S^E^), for the most promising FA derivatives.

According to the gathered results (Figure 2), toxicity is responsible for the largest deviation. Regarding ADME, the six additional descriptors lead to the largest deviation than logP and MW. Synthetic accessibility also has a rather small influence on the deviations from the reference molecules. A more detailed examination, considering the individual contribution of all the investigated descriptors, is presented in Figure 3. 

The more important deviations arise from LD_50_, followed by M, PSA and HB^D^. The FA derivatives with the largest LD_50_ deviations from the reference set are FA, FA-173, FA-175 and FA-26. However, they correspond to a lower toxicity to rats than the average of the reference set (LD_50_ = 960.8), with values of 4742.7, 4471.9, 4040.7, and 3635.2, respectively. Regarding Ames mutagenicity, a similar trend was found. The FA derivatives predicted as the least mutagenic are just those that deviate the most from the reference set average (M = 0.41). They are FA-88, FA-106, FA-115 and FA-142, all with M = 0.01. Thus, these deviations imply that the above-mentioned derivatives have a more desirable behavior than that of the reference set. Accordingly, they were not excluded from the chosen subset. 

The largest PSA deviation were found for FA-41, FA-26, FA-88 and FA-173 (124.3, 113.0, 104.1 and 104.1 Å^2^, respectively). However, their PSA values are all below Veber’s limit: 140 Å^2^. Thus, these derivatives were also kept in the chosen subset. The largest deviations for HB^D^ correspond to FA-26 with HB^D^ = 5 and FA-8, FA-41 and FA-138 with HB^D^ = 4. Since they do not represent violations of Lipinski’s rule, these candidates were not eliminated. 

After carefully examining elimination scores for the 12 FA derivatives with the highest S^S^ values, none of them were excluded from the selection. Thus, they were investigated regarding their antioxidant capacity through electron and H-atom donation. This detailed analysis is important since it allows interpreting deviations for all the used descriptors and prevents the exclusion of suitable candidates for no good reason. 

### 3.2. pKa and Antioxidant Activity

As previously mentioned, acid-base equilibria are crucial for medical drugs intended to passively cross biological barriers. The *p*Ka values and molar fractions (^M^*f*) at physiological pH were estimated for the 12 FA derivatives chosen in the first stage of the investigation as those with the best drug-like behavior (Table 2). Additionally, the corresponding deprotonation routes and distribution diagrams are provided in Figures S2 and S3. 

The calculated molar fractions (Table 2) revealed that 7 of the 12 derivatives, selected based on the S^S^ value, would have a negligible population (<10^−4^) at physiological pH, i.e., at pH = 7.4. Thus, they were excluded as viable candidates. Although the ^M^*f*_(0)_ for the other three (FA-26, FA-118, and FA-175) are rather small, they are very similar to that of FA. Since there is abundant data on the biological activities of FA (Table 1), it can be inferred that such fractions are enough. Consequently, five derivatives (FA-8, FA-26, FA-118, FA-138, and FA-175) were further investigated. Among the studied derivatives, FA-138 is the only one that is predicted to have similar fractions of neutral (q = 0) and anionic (q = −1) species. This feature might be relevant to its possible use as a multifunctional antioxidant. The rather large neutral fraction (59.0%) is expected to promote passive crossing through biological membranes, while the anionic fraction (40.3%) is likely to be the key one for the free radical scavenging activity, as it is the case for many phenolic compounds. 

The ionization energies (IE), electron affinities (EA), and the lowest bond dissociation energies (BDE) for the acid-base species with a non-negligible population (^M^*f*_(q)_ ≥ 10^−4^) of FA and its derivatives at pH = 7.4, are reported in Table 3. The complete set of BDEs, i.e., considering all viable H-donating sites, is provided as Supplementary Materials (Table S8). IE and BDE reactivity indexes are related to the viability of electron and H-atom donation. Thus, they were used to compare the efficiency of the derivatives with that of reference antioxidants as free radical scavengers via single electron transfer (SET) and formal hydrogen atom transfer (HAT) mechanisms, respectively.

**Figure 4:**
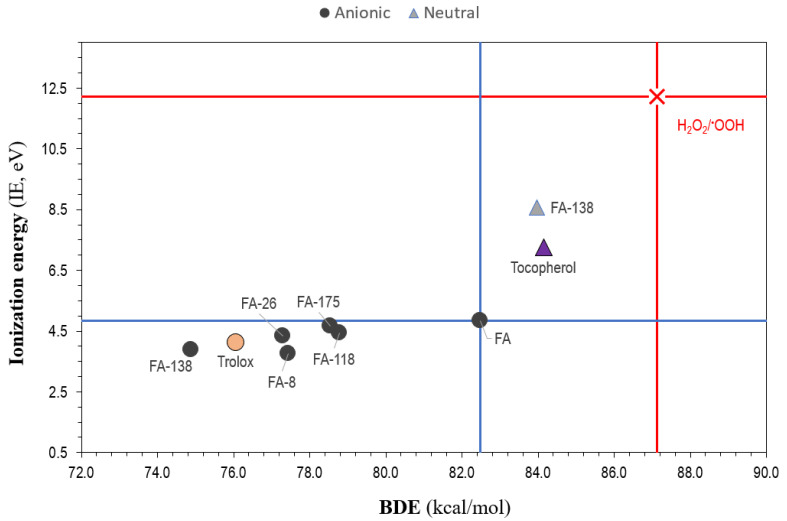
The electron and hydrogen donating ability map for antioxidants (eH-DAMA), including the dominant acid-base species of FA derivatives, the parent molecule, Trolox, α-tocopherol, and the H_2_O_2_/O_2_^•−^ oxidant pair.

IE and BDE values were used to build the electron and hydrogen-donating ability map for antioxidants (eH-DAMA, Figure 4). This graphical tool has been recently proposed to simultaneously account for the likeliness of molecules as H donors (formal HAT reaction route) and electron donors (SET reaction route) [92,93]. The dominant acid-base species of the investigated FA derivatives at physiological pH were included in this map, as well as two antioxidant references (Trolox and α-tocopherol), the parent molecule, and the H_2_O_2_/O_2_^•−^. This pair represents the potential oxidant target. The best radical scavengers are expected to be located at the bottom left, i.e., lower IE and lower BDE. The species in this region are likely to simultaneously act as electron and H-atom donors.

Based on the eH-DAMA (Figure 4), it is predicted that the five FA derivatives included in it should be efficient for scavenging peroxyl radicals through both mechanisms, SET and *f*-HAT. Their efficiency for that purpose is expected to surpass that of α-tocopherol and ferulic acid. On the contrary, only the anionic form of FA-138 is predicted to be more efficient than Trolox for that purpose. FA-8 may be a better electron donor than Trolox but not as good for donating H-atoms. However, further investigations dealing with other aspects of antioxidant activity, kinetics in particular, are still needed to confirm or refute the foreseen trends. 

### 3.3. Polygenic Activity

To evaluate general neuroprotection activity, a polygenic score (S_P_) was developed. S_P_ is a measure of the tested compounds' capacity to bind to the enzymes compared with natural substrates (COMT: dopamine (dopa), MAO-B phenylethylamine (pea) and AChE: acetylcholine (ACh). It was defined according to our previous reports [136,137] as:

$$S_{P}=\frac{{∆G}_{B,COMT}^{W}}{{∆G}_{B,dopa}}+\frac{{∆G}_{B,MAO-B}^{W}}{{∆G}_{B,pea}}+\frac{{∆G}_{B,AChE}^{W}}{{∆G}_{B,ACh}}$$

The scoring values are presented in Table 4. When the values of S_P_ are examined, it can be predicted that the compounds exhibit neuroprotection activity since their scores are higher than those of the corresponding natural substrates (S_P_ = 3.00), i.e., the investigated ferulic acid derivatives may present stronger affinities towards the enzymes. Among the studied compounds, the FA-26 analog is expected to have the best neuroprotection activity. Interestingly, according to the docking results, the parent molecule (ferulic acid) is also likely to act as a neuroprotector. 

**Figure 5:**
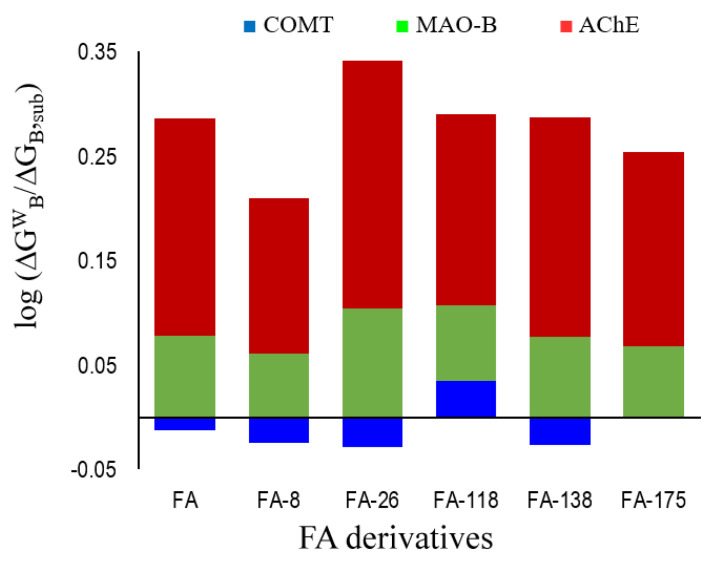
Polygenic scores of the ferulic acid and its derivatives.

The examination of individual ΔG_B_^W^ values reveals that the studied compounds could be better inhibitors for AChE and MAO-B than they are for the COMT enzyme. Negative values of COMT (blue fragment of the bars in Figure 5) indicate that this enzyme forms more stable complexes with dopamine than with the tested FA derivatives. Only FA-118 shows a slightly higher score than dopamine. Interestingly, this compound has a catechol moiety, which is recognized to exhibit effective COMT inhibition potential [187]. FA-175 presents almost the same score as dopamine (log ΔG^W^_B_/ΔG_B_,_sub_= −0.001). On the other hand, for MAO-B and AChE (green and red fragments, respectively, Figure 5), the neuroprotection behavior of FA derivatives was evidenced by their positive values. Between these two enzymes, the inhibitor potential of the studied derivatives is expected to be stronger for AChE. The binding energies ΔG_B_ values per acid-base species of the most promised derivatives can be found in Table S9, and the complete set of ΔG_B_^W^ for the thirteen selected derivatives (see Scheme 2) can be consulted in Table S10, Supplementary Materials.

**Figure 6:**
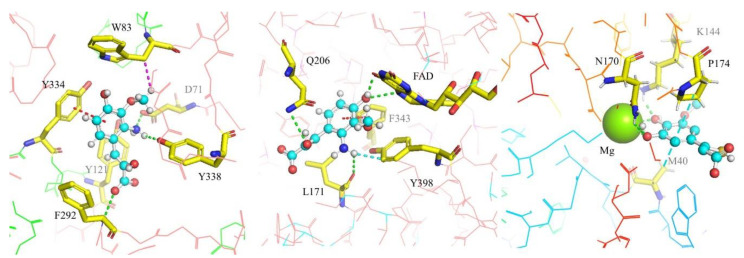
Interactions in FA-26:AChE (**left**), FA-26:MAOB (**middle**) and FA-118:COMT complexes (**right**). FA-26 and FA-118 are shown using the ball and stick model. Interactions are presented as dotted lines: conventional hydrogen bonds (green), π-stacking (red), π-alkyl (magenta), and C-H non-conventional bonds (cyan).

Molecular docking allows the prediction of the binding conformations between therapeutic targets and small molecules. The analysis of the possible interactions that form the adducts promotes development and drug discovery. We must not lose sight of the limitations of the method, and if the work demands obtaining more realistic conformations, the use of more precise tools such as molecular dynamics or QM protocols is essential. Even so, molecular docking has been shown to provide reliable predictions of non-covalent bonds, such as hydrogen bonding and hydrophobic interactions [187,188]. The main interactions for the complexes with the highest S_P_ are shown in Figure 6. They are FA-26 with AChE (left), FA-26 with MAO-B (middle), and FA-118 with COMT (right). For all of them, FA-26 is in its anionic form, which is the most abundant species at physiological pH (X ~ 0.97). To understand the interactions formed in the protein-ligand complexes, it is important to know the architecture of the enzymes and the function of the key residues. AChE has a highly specialized structure, which allows it to be one of the fastest-known enzymes. The catalytic triad (H447, E334 and S203) is found at the bottom of the enzyme and surrounded by 14 well-conserved aromatic residues [189]. Among them, W83 plays an essential role since it forms a substrate union site, while Y70, Y121, and W279 conform to the anionic peripheric site [189]. Additionally, AChE has a high dipole moment with the axis oriented towards the substrate entry site. It has been suggested that this moment may serve to pull down the cationic substrate of AChE. This dipole is controlled mainly by residues D71, E199, and E443 [190]. The binding and anionic sites are responsible for supporting the cationic substrate acetylcholine by the ammonium group, as well as both quaternary ligands (edrophonium, N-methylacridinium) acting as competitive inhibitors. In the catalytic site, the ester hydrolysis leads to the formation of an acyl group attached to the enzyme and the release of choline. Then, a water molecule assists at residue H447, releasing acetic acid, regenerating free enzyme and ending the function of this neurotransmitter [191]. The pharmacological effect of AChE inhibitors consists of the inactivation of the enzymatic activity resulting in the increase of synaptic ACh and the stimulation of postsynaptic cholinergic receptors in the central and peripheral nervous systems. Therefore, these drugs improve cholinergic neurotransmission and compensate for the loss of brain cells in some conditions, such as Alzheimer’s, providing benefits in all the key symptoms of the disease [192]. 

FA-26 has several H-bond donors and acceptors and an aromatic ring that contributes to generating intermolecular connections with the AChE key amino acids. In fact, complex FA-26:AChE is formed by several interactions, mainly hydrogen bonds and π-interactions. This derivative is bonded to the active site of AChE through four hydrogen bonds (D71, Y121, F292, and Y338), one π-stacking interaction (Y334) and one p-alkyl interaction (W83). The observed interactions suggest that FA-26, although not bonded to the catalytic triad, can inhibit ACh degradation, blocking the entry and union sites.

MAO-B function involves two hydrophobic pockets, an entry pocket and an active site pocket, with I199 acting as a gatekeeper between two cavities. The catalytic reaction site comprises a redox cofactor, flavin adenine dinucleotide (FAD). The active site is completed by residues Y398 and Y435, orienting the substrate to the proper position [193]. The enzyme promotes the oxidation of amines, generating aldehyde, ammonia and hydrogen peroxide. Although the mechanism has not been fully elucidated, studies with MAO inhibitors suggest that FAD is a key fragment in the transformation of amines [194]. Inhibitors of MAO-B are used to conserve adequate levels of several neurotransmitters as dopamine, norepinephrine, and serotonin, or to increase them. For this reason, MAO-B inhibitors are used to treat depression and alleviate the symptoms of Parkinson’s disease [195].

Four H-bonds involving Q206, L171, and FAD, a p-stacking (F343) and non-conventional C-H bonds stabilize the complex formation. An important feature of the conformation adopted by FA-26 in the complex is the formation of an H-bond with N5 in the FAD moiety. This atom is required for the redox activity of the cofactor [193] and, hence, for the catalytic function of the enzyme. This conformation could not be achieved without the orientation promoted by the L171 and Y398 residues, which suggests that FA-26 could inhibit some enzymes with the same mechanism of action as MAO (type A) or other flavoenzymes as lactate oxidase [196]. According to these findings, FA-26 is predicted to act as a reversible or non-covalent MAO-B inhibitor as Safinamide or Moclobemide [175,197], which are recognized antidepressant drugs. This way of inhibition is preferable since it has been proven to be associated with less toxicity than others [198]. 

COMT is a selective enzyme that catalyzes the transfer of methyl groups to the 3-OH position of catecholamines. COMT is an Mg-dependent enzyme, with the metal bound to D141, D169, and N170 residues. This enzyme uses the Mg atom to bind the substrate and make it more easily ionizable [199]. The methyl group is transferred by the S-Adenosylmethionine cofactor. The binding substrate site is completed with several hydrophobic residues M40, L198, W143, and the gatekeepers W38 and P174 [199]. COMT is responsible for the selective methylation of catecholamines hydroxyls, including dopamine, epinephrine, and norepinephrine. The inhibition of this protein has become a key strategy to manipulate the levels of these neurotransmitters and other substances that are dopamine precursors, such as L-DOPA or Carbidopa, used to treat Parkinson's disease [187]. 

According to the docking simulations, FA-118 has a catechol moiety that binds the Mg atom by two metal-donor unions. A hard acid-base interaction (Mg-O) stabilizes the formation of this adduct. In addition, H-bonds between the catechol fragment and the K144 and N170 residues also contribute to the binding energy. Finally, several hydrophobic interactions with key residues of the active site (M40 and P174) complete the stabilization of the FA-118:COMT complex. Such an arrangement explains the good score obtained in the simulations and suggests that FA-118 can be efficient as a COMT inhibitor. 

The docking simulations indicate that while all the investigated FA derivatives can act as neuroprotectors of acetylcholine and phenylethylamine (with FA-26 being predicted as the best one for that purpose), only FA-118 would be able to protect dopamine against COMT-induced degradation. Accordingly, FA-118 is proposed as a promising candidate in the context of Alzheimer’s and/or anti-anxiety disorders, while FA-26 was identified as the best candidate (among the studied molecule) for Parkinson’s. All of them certainly deserve further investigations related to their potential as neuroprotectors.

## 4. Conclusions

A total of 185 ferulic acid (FA) derivatives were built through a rational in silico design using the CADMA-Chem protocol. The chemical space was sampled using a selection score (S^S^) that considers ADME properties, toxicity and synthetic accessibility descriptors. Based on the estimated S^S^ values, 12 FA derivatives were identified as the candidates with the best drug-like behavior. For this subset, some reactivity indexes were computed, as well as their pKa values. According to eH-DAMA results, which take into account the free radical scavenging behavior through single electron transfer (SET) and formal hydrogen transfer (HAT) mechanisms, FA-138 seems to be the best candidate to scavenge free radicals. However, FA-8, FA-26, FA-118, and FA-175 derivatives are predicted to be better for that purpose than α-tocopherol and the parent molecule. 

On the other hand, docking studies suggest that ferulic acid and some of its derivatives can act as inhibitors of AChE and MAO-B enzymes. FA-26 is predicted as the most efficient one for that purpose. This compound is bound preferably to the entry site of AChE and to the catalytic site of MAO-B, acting as a reversible inhibitor for the latter. On the contrary, FA-118 was the only compound identified as a viable candidate to efficiently inhibit COMT. Accordingly, FA-26 is proposed as the best candidate in the context of Alzheimer’s and/or anti-anxiety disorders and FA-118 for Parkinson’s. At least these two compounds certainly deserve further investigation regarding their potential role as neuroprotectors.

Considering the gathered data altogether, the FA derivatives proposed for further investigations are FA-26, FA-118, and FA-138. 

## Figures and Tables

**Table 1:** Structural modifications and properties of some FA derivatives.

Functionalization	Bioactivity	Ref.
3-n-butylphthalide + glucose	Anti-ischemic.	[123]
Alkyl esters	β-amyloid aggregation inhibition.	[124]
Amide	Antiviral	[127]
Amide	Antioxidant, inflammatory, mitophagy enhancing.	[126]
Amide	β-amyloid oligomerization and fibrillization inhibition.	[103]
Amide	Antioxidant, anticancer.	[27]
Amide + pyrazole	Antioxidant and myocardial cell hypoxia reoxygenation.	[133]
Amino acid	Anti-inflammatory, antioxidant.	[110]
Aniline	Antimicrobial.	[101]
Azetidine-2-one	Anti-inflammatory, antioxidant.	[96,97]
Benzyl and phenylethyl esters	Anticancer.	[29]
Benzylamino, and carbamyl	β-amyloid aggregation inhibition, antioxidant, AChE inhibition.	[105]
Cyclized	Antiviral.	[134]
Different rings	Improvement of scopolamine-induced memory deficit in mice.	[116]
Dimer	Neuroprotection.	[100]
Dimethylthiazol + diphenyltetrazolium bromide	Anticancer.	[129]
Ester	Antibacterial.	[113,114]
Ester	Antifungal.	[131]
Ester	Antithrombotic.	[130]
Ester	Anticancer.	[107]
Ester	Xanthine oxidase inhibition.	[121]
Ester and amide	Anticancer.	[118]
Ester and amide	Antioxidant.	[90]
Glycerol and diglycerol	β-amyloid aggregation inhibition.	[102]
Heterocyclic	Anticancer.	[109]
Isopentyl	Anticonvulsant.	[135]
N-Hydroxy-N-Propargylamide	Free radical scavenging, AChE inhibition, Cu(II) quelation.	[92]
O-alkylamines	Antioxidant, butyrylcholinesterase inhibition.	[115]
OH + OMe group + amide	Neuraminidase inhibition.	[94]
Phthalate and maleate	Hepatoprotection.	[91]
Piperazine	Antiviral.	[128]
Tributyltin(IV)	Anticancer.	[132]

**Table 2:** Estimated *p*Ka values and molar fractions, ^M^*f*_(q)_, at pH = 7.4. The (q) in the acronym represents the charge of the acid-base species.

	*p*Ka_1_	*p*Ka_2_	*p*Ka_3_	*p*Ka_4_	^M^ *f*_(+1)_	^M^ *f*_(0)_	^M^ *f*_(−1)_	^M^ *f*_(−2)_	^M^ *f*_(−3)_	^M^ *f*_(−4)_
FA	4.0	10.0	-	-	-	4 × 10^−4^	0.997	0.003	-	-
FA-8	3.1	4.4	5.8	10.8	<10^−4^	0.023	0.976	4 × 10^−4^	-	-
FA-12	3.7	5.6	10.3	11.3	-	<10^−4^	0.015	0.984	0.001	<10^−4^
FA-26	2.5	3.9	10.1	11.8	<10^−4^	3×10^−4^	0.998	0.002	<10^−4^	-
FA-41	2.7	4.4	9.7	11.1	-	<10^−4^	0.001	0.994	0.005	<10^−4^
FA-88	1.9	5.2	5.9	10.5	-	<10^−4^	2 × 10^−4^	0.033	0.966	0.001
FA-106	3.7	5.7	10.3	11.2	-	<10^−4^	0.019	0.980	0.001	<10^−4^
FA-115	3.8	5.8	8.6	10.9	-	<10^−4^	0.023	0.918	0.059	<10^−4^
FA-118	3.6	8.5	9.5	13.0	-	1 × 10^−4^	0.921	0.078	0.001	<10^−4^
FA-138	4.0	7.6	9.9	-	2 × 10^−4^	0.596	0.403	0.001	-	-
FA-142	3.1	5.8	10.4	12.2	-	<10^−4^	0.022	0.977	0.001	<10^−4^
FA-173	3.6	4.2	10.0	-	-	<10^−4^	0.001	0.997	0.003	-
FA-175	3.8	9.6	11.0	-	-	3 × 10^−4^	0.994	0.006	<10^−4^	-

**Table 3:** First ionization energy (IE, eV), electron affinities (EA, eV), and lowest bond dissociation energies (BDE, kcal/mol) for FA and the selected subset of derivatives.

	IE	EA	BDE	BDE-Site *
q = 1				
FA-138	11.64	3.54	89.30	b (OH)
q = 0				
FA	8.36	−0.28	85.15	b (OH)
FA-8	8.75	−0.13	80.24	R5 (SH)
FA-26	8.35	0.34	83.06	R1 (OH)
FA-118	8.31	−0.95	80.18	b (OH)
FA-138	8.58	0.29	83.97	b (OH)
FA-175	8.12	−0.23	80.09	R2 (OH)
q = −1				
FA	4.85	−2.95	82.48	b (OH)
FA-8	3.75	−2.95	77.44	R5 (SH)
FA-26	4.33	−3.12	77.29	R1 (OH)
FA-118	4.43	−2.98	78.79	b (OH)
FA-138	3.89	−3.11	74.88	b (OH)
FA-175	4.66	−3.05	78.54	b (OH)
q = −2				
FA	−0.06	−6.00	96.96	a (OCH_3_)
FA-8	0.20	−5.58	82.04	b (OH)
FA-26	−0.34	−6.04	71.30	b (OH)
FA-118	−1.09	−4.97	74.87	b (OH)
FA-138	−0.55	−5.77	97.02	a (OCH_3_)
FA-175	−0.31	−5.32	75.90	b (OH)
q= −3				
FA-118	−3.78	−7.59	71.01	b (OH)

* The labels correspond to those shown in Scheme 1.

**Table 4:** Polygenic score (S_P_) values for ferulic acid and its derivatives.

Compound	DG_B_^W^ (kcal/mol)			S_P_
	COMT	MAO-B	AChE	
**Ferulic Acid**	−5.28	−7.19	−7.37	3.78
FA-9	−5.14	−6.91	−6.43	3.50
FA-26	−5.09	−7.63	−7.88	3.93
FA-118	−5.90	−7.09	−6.95	3.79
FA-138	−5.12	−7.17	−7.40	3.76
FA-175	−5.42	−7.02	−7.01	3.70

ΔG_B_, _dopa_ = −5.44 kcal/mol in COMT; ΔG_B_, _pea_ = −6.01 kcal/mol in MAO-B; ΔG_B_, _Ach_ = 4.56 kcal/mol in AChE. For natural substrates S_P_ = 3.00.

## Data Availability

Data is contained within the article and supplementary materials.

## Supplementary Materials

The following supporting information can be downloaded at: https://www.mdpi.com/article/10.3390/antiox12061256/s1. Table S1: FA derivatives designed in this work. Table S2: Values of the ADME properties, toxicity descriptors, synthetic accessibility, and selection score (S^S^) for all designed derivatives. Table S3: Elimination scores for the subset of ferulic acid derivatives chosen as the most promising, according to S^S^. Table S4: Reference set of molecules with some neuroprotective effects. Table S5: Pole strength values for the EPT approximation (P3) used to calculate ionization energies and electron affinities. Table S6: Equations concerning S^S^ construction. Table S7: Exclusion scores (S^E^) equations. Table S8: Complete set of BDEs for ferulic acid and its derivatives. Table S9: Complete set of the binding energies for ferulic acid and its derivatives. Figure S2: Redocking simulation: tolcapone in COMT, Safrinamide in MAO-B, and Donopezil in AChE. Figure S3: Deprotonation routes for the subset of ferulic acid derivatives chosen as the most promising from their drug-like behavior. Figure S4: Distribution diagram of the acid-base species of ferulic acid derivatives.
