# Supplementary material for: Rational Design of Multifunctional Ferulic Acid Derivatives Aimed for Alzheimer’s and Parkinson’s Diseases
Source: Antioxidants (Basel). 2023 Jun 11;12(6):1256. doi: 10.3390/antiox12061256 (PMC10295297; doi:10.3390/antiox12061256)
Supplement: Supplementary file 1 [file antioxidants-12-01256-s001.zip › antioxidants-2399315-supplementary.pdf]

Supplementary Materials

Rational Design of Multifunctional Ferulic Acid Derivatives Aimed for Alzheimer's and Parkinson's diseases

Eduardo Gabriel Guzmán-López,[1] Miguel Reina,[2] Luis Felipe Hernandez-Ayala,[1] and Annia Galano[1*]

[1] *Departamento de Química, Universidad Autónoma Metropolitana-Iztapalapa, Av. Ferrocarril San Rafael Atlixco 186, Col. Leyes de Reforma 1A Sección, Alcaldía Iztapalapa, Mexico City 09310, Mexico.*

[2] *Departamento de Química Inorgánica y Nuclear, Facultad de Química, Universidad Nacional Autónoma de México, Mexico City 04510, Mexico.*

Contents

Table S1. Ferulic acid (FA) derivatives designed in this work. 3

Table S2. Values of the ADME properties: water/octanol partition coefficient (logP), topological polar surface area (PSA), number of heavy atoms (^{X}At), molecular weight (MW), number of H-bond acceptors (HBA), number of H-bond donors (HBD), rotatable bonds (RB), and molar refractivity (MR); toxicity descriptors: oral rat 50 percent lethal dose (LD50); Ames' mutagenicity (M); the synthetic accessibility (SA); and selection score (S^S).. 8

Table S3. Elimination scores for the subset of ferulic acid derivatives chosen as the most promising, according to S^S. 14

Table S4. Reference set of molecules, with some neuroprotective effects. 15

Table S5. Pole strength (PS) values for the EPT approximation (P3) used to calculated ionization energies and electron affinities. 17

Table S6. Equations concerning S^S construction. 18

Table S7. Exclusion scores (S^E) equations. 19

Table S8. Zero-point bond dissociation energies (BDE, in kcal/mol) for ferulic acid and its derivatives. Only the species with molar fractions ≥10^{-4} are included................................ 20

Table S9. Binding energies (ΔG_B, kcal/mol) for acid-base species of ferulic acid and its derivatives.. 21

Table S10. Complete set of weighted binding energies ($\Delta G^w{}_B$, kcal/mol) for FA and its derivatives.. 22

Figure S1. Correlation between pKa values estimated with Marvin software vs experimentally pKa values. 23

Figure S2. Redocking simulations of tolcapone in comt (left), Safrinamide in MAO-B (center) and Donopezil in AChE (right).. 24

Figure S3. Deprotonation routes for the subset of ferulic acid derivatives chosen as the most promising, from their drug-like behavior. 25

Figure S4. Distribution diagram of the acid-base species of ferulic acid derivatives. The vertical line landmarks the physiological pH (pH=7.4). 26

Table S1. Ferulic acid (FA) derivatives designed in this work.

	R_1	R_2	R_3	R_4	R_5
FA	H	H	H	H	H
FA-1	H	H	H	COOH	COOH
FA-2	H	H	H	COOH	NH2
FA-3	H	H	H	COOH	OH
FA-4	H	H	H	COOH	SH
FA-5	H	H	H	NH2	COOH
FA-6	H	H	H	NH2	NH2
FA-7	H	H	H	NH2	OH
FA-8	H	H	H	NH2	SH
FA-9	H	H	H	OH	COOH
FA-10	H	H	H	OH	NH2
FA-11	H	H	H	OH	OH
FA-12	H	H	H	OH	SH
FA-13	H	H	H	SH	COOH
FA-14	H	H	H	SH	NH2
FA-15	H	H	H	SH	OH
FA-16	H	H	H	SH	SH
FA-17	COOH	H	H	H	COOH
FA-18	COOH	H	H	H	NH2
FA-19	COOH	H	H	H	OH
FA-20	COOH	H	H	H	SH
FA-21	NH2	H	H	H	COOH
FA-22	NH2	H	H	H	NH2
FA-23	NH2	H	H	H	OH
FA-24	NH2	H	H	H	SH
FA-25	OH	H	H	H	COOH
FA-26	OH	H	H	H	NH2
FA-27	OH	H	H	H	OH
FA-28	OH	H	H	H	SH
FA-29	SH	H	H	H	COOH
FA-30	SH	H	H	H	NH2
FA-31	SH	H	H	H	OH
FA-32	SH	H	H	H	SH
FA-33	H	COOH	H	H	COOH
FA-34	H	COOH	H	H	NH2
FA-35	H	COOH	H	H	OH
FA-36	H	COOH	H	H	SH
FA-37	H	NH2	H	H	COOH
FA-38	H	NH2	H	H	NH2
FA-39	H	NH2	H	H	OH

FA-40	H	NH2	H	H	SH
FA-41	H	OH	H	H	COOH
FA-42	H	OH	H	H	NH2
FA-43	H	OH	H	H	OH
FA-44	H	OH	H	H	SH
FA-45	H	SH	H	H	COOH
FA-46	H	SH	H	H	NH2
FA-47	H	SH	H	H	OH
FA-48	H	SH	H	H	SH
FA-49	H	H	COOH	H	COOH
FA-50	H	H	COOH	H	NH2
FA-51	H	H	COOH	H	OH
FA-52	H	H	COOH	H	SH
FA-53	H	H	NH2	H	COOH
FA-54	H	H	NH2	H	NH2
FA-55	H	H	NH2	H	OH
FA-56	H	H	NH2	H	SH
FA-57	H	H	OH	H	COOH
FA-58	H	H	OH	H	NH2
FA-59	H	H	OH	H	OH
FA-60	H	H	OH	H	SH
FA-61	H	H	SH	H	COOH
FA-62	H	H	SH	H	NH2
FA-63	H	H	SH	H	OH
FA-64	H	H	SH	H	SH
FA-65	H	H	H	H	COOH
FA-66	H	H	H	H	NH2
FA-67	H	H	H	H	OH
FA-68	H	H	H	H	SH
FA-69	COOH	H	H	COOH	H
FA-70	COOH	H	H	NH2	H
FA-71	COOH	H	H	OH	H
FA-72	COOH	H	H	SH	H
FA-73	NH2	H	H	COOH	H
FA-74	NH2	H	H	NH2	H
FA-75	NH2	H	H	OH	H
FA-76	NH2	H	H	SH	H
FA-77	OH	H	H	COOH	H
FA-78	OH	H	H	NH2	H
FA-79	OH	H	H	OH	H
FA-80	OH	H	H	SH	H
FA-81	SH	H	H	COOH	H
FA-82	SH	H	H	NH2	H

FA-83	SH	H	H	OH	H
FA-84	SH	H	H	SH	H
FA-85	H	COOH	H	COOH	H
FA-86	H	NH2	H	COOH	H
FA-87	H	OH	H	COOH	H
FA-88	H	SH	H	COOH	H
FA-89	H	H	COOH	COOH	H
FA-90	H	H	NH2	COOH	H
FA-91	H	H	OH	COOH	H
FA-92	H	H	SH	COOH	H
FA-93	H	H	H	COOH	H
FA-94	H	COOH	H	NH2	H
FA-95	H	NH2	H	NH2	H
FA-96	H	OH	H	NH2	H
FA-97	H	SH	H	NH2	H
FA-98	H	H	COOH	NH2	H
FA-99	H	H	NH2	NH2	H
FA-100	H	H	OH	NH2	H
FA-101	H	H	SH	NH2	H
FA-102	H	H	H	NH2	H
FA-103	H	COOH	H	OH	H
FA-104	H	NH2	H	OH	H
FA-105	H	OH	H	OH	H
FA-106	H	SH	H	OH	H
FA-107	H	H	COOH	OH	H
FA-108	H	H	NH2	OH	H
FA-109	H	H	OH	OH	H
FA-110	H	H	SH	OH	H
FA-111	H	H	H	OH	H
FA-112	H	COOH	H	SH	H
FA-113	H	NH2	H	SH	H
FA-114	H	OH	H	SH	H
FA-115	H	SH	H	SH	H
FA-116	H	H	COOH	SH	H
FA-117	H	H	NH2	SH	H
FA-118	H	H	OH	SH	H
FA-119	H	H	SH	SH	H
FA-120	H	H	H	SH	H
FA-121	COOH	COOH	H	H	H
FA-122	COOH	NH2	H	H	H
FA-123	COOH	OH	H	H	H
FA-124	COOH	SH	H	H	H
FA-125	COOH	H	COOH	H	H

FA-126	COOH	H	NH2	H	H
FA-127	COOH	H	OH	H	H
FA-128	COOH	H	SH	H	H
FA-129	COOH	H	H	H	H
FA-130	NH2	COOH	H	H	H
FA-131	NH2	NH2	H	H	H
FA-132	NH2	OH	H	H	H
FA-133	NH2	SH	H	H	H
FA-134	NH2	H	COOH	H	H
FA-135	NH2	H	NH2	H	H
FA-136	NH2	H	OH	H	H
FA-137	NH2	H	SH	H	H
FA-138	NH2	H	H	H	H
FA-139	OH	COOH	H	H	H
FA-140	OH	NH2	H	H	H
FA-141	OH	OH	H	H	H
FA-142	OH	SH	H	H	H
FA-143	OH	H	COOH	H	H
FA-144	OH	H	NH2	H	H
FA-145	OH	H	OH	H	H
FA-146	OH	H	SH	H	H
FA-147	OH	H	H	H	H
FA-148	SH	COOH	H	H	H
FA-149	SH	NH2	H	H	H
FA-150	SH	OH	H	H	H
FA-151	SH	SH	H	H	H
FA-152	SH	H	COOH	H	H
FA-153	SH	H	NH2	H	H
FA-154	SH	H	OH	H	H
FA-155	SH	H	SH	H	H
FA-156	SH	H	H	H	H
FA-157	H	COOH	COOH	H	H
FA-158	H	NH2	COOH	H	H
FA-159	H	OH	COOH	H	H
FA-160	H	SH	COOH	H	H
FA-161	H	COOH	NH2	H	H
FA-162	H	NH2	NH2	H	H
FA-163	H	OH	NH2	H	H
FA-164	H	SH	NH2	H	H
FA-165	H	COOH	OH	H	H
FA-166	H	NH2	OH	H	H
FA-167	H	OH	OH	H	H
FA-168	H	SH	OH	H	H

FA-169	H	COOH	SH	H	H
FA-170	H	NH2	SH	H	H
FA-171	H	OH	SH	H	H
FA-172	H	SH	SH	H	H
FA-173	H	COOH	H	H	H
FA-174	H	NH2	H	H	H
FA-175	H	OH	H	H	H
FA-176	H	SH	H	H	H
FA-177	H	H	COOH	H	H
FA-178	H	H	NH2	H	H
FA-179	H	H	OH	H	H
FA-180	H	H	SH	H	H

Table S2. Values of the ADME properties: water/octanol partition coefficient (logP), topological polar surface area (PSA), number of heavy atoms (^{X}At), molecular weight (MW), number of H-bond acceptors (HBA), number of H-bond donors (HBD), rotatable bonds (RB), and molar refractivity (MR); toxicity descriptors: oral rat 50 percent lethal dose (LD50); Ames' mutagenicity (M); the synthetic accessibility (SA); and selection score (S^{S}).

	logP	PSA	^{x}At	MW	HB^{A}	HB^{D}	RB	^{M}R	LD_{50}	M	SA	S^{S}
FA	1.25	66.76	14	194.19	4	2	3	51.33	4742.73	0.22	2.13	3.77
FA-1	-0.30	141.36	20	282.20	8	4	5	64.87	1672.76	0.52	3.72	2.99
FA-2	-0.39	130.08	18	253.21	7	5	4	62.32	2289.44	0.33	3.30	3.34
FA-3	-0.10	124.29	18	254.19	7	4	4	59.57	1181.06	0.28	3.26	3.23
FA-4	0.35	104.06	18	270.26	6	3	4	65.16	1003.90	0.16	3.32	3.31
FA-5	-0.15	130.08	18	253.21	7	5	4	61.42	3936.05	0.18	3.51	3.56
FA-6	-0.24	118.81	16	224.22	6	6	3	58.87	2458.08	0.41	3.12	3.20
FA-7	0.06	113.01	16	225.20	6	5	3	56.12	1117.37	0.18	3.09	3.34
FA-8	0.51	92.78	16	241.27	5	4	3	61.71	1911.70	0.04	3.15	3.78
FA-9	1.21	124.29	18	254.19	7	4	4	59.86	3182.26	0.59	3.41	3.27
FA-10	1.12	113.01	16	225.20	6	5	3	57.31	1101.01	0.14	3.01	3.40
FA-11	1.41	107.22	16	226.18	6	4	3	54.57	1964.96	0.33	2.98	3.35
FA-12	1.86	86.99	16	242.25	5	3	3	60.15	2424.68	0.04	3.03	3.85
FA-13	0.76	104.06	18	270.26	6	3	4	66.22	1213.44	0.29	3.51	3.20
FA-14	0.67	92.78	16	241.27	5	4	3	63.67	1358.57	0.21	3.13	3.35
FA-15	0.97	86.99	16	242.25	5	3	3	60.92	2485.60	0.23	3.09	3.46
FA-16	1.42	66.76	16	258.32	4	2	3	66.51	2034.75	0.28	3.15	3.37
FA-17	-0.05	141.36	20	282.20	8	4	5	64.87	3249.58	0.43	3.63	3.19
FA-18	-0.14	130.08	18	253.21	7	5	4	62.32	1928.03	0.64	3.23	3.16
FA-19	0.16	124.29	18	254.19	7	4	4	59.57	2351.06	0.27	3.15	3.41
FA-21	0.29	130.08	18	253.21	7	5	4	61.42	4091.49	0.25	3.48	3.50
FA-22	0.20	118.81	16	224.22	6	6	3	58.87	3023.67	0.19	3.09	3.42
FA-23	0.49	113.01	16	225.20	6	5	3	56.12	2352.39	0.25	3.06	3.44

FA-24	0.94	92.78	16	241.27	5	4	3	61.71	1979.75	0.08	3.12	3.64
FA-25	0.43	124.29	18	254.19	7	4	4	59.86	3493.74	0.36	3.48	3.39
FA-26	0.34	113.01	16	225.20	6	5	3	57.31	3635.20	0.11	3.09	3.70
FA-27	0.64	107.22	16	226.18	6	4	3	54.57	3011.62	0.20	3.05	3.54
FA-28	1.09	86.99	16	242.25	5	3	3	60.15	1506.13	0.07	3.12	3.61
FA-29	0.82	104.06	18	270.26	6	3	4	66.22	3312.19	0.30	3.48	3.41
FA-30	0.73	92.78	16	241.27	5	4	3	63.67	3573.17	0.48	3.09	3.38
FA-31	1.03	86.99	16	242.25	5	3	3	60.92	1807.44	0.56	3.06	3.20
FA-32	1.47	66.76	16	258.32	4	2	3	66.51	1312.43	0.25	3.12	3.30
FA-33	0.27	141.36	20	282.20	8	4	5	65.25	3646.68	0.07	3.68	3.60
FA-34	0.18	130.08	18	253.21	7	5	4	62.70	3807.34	0.19	3.42	3.55
FA-35	0.48	124.29	18	254.19	7	4	4	59.95	3903.17	0.22	3.37	3.53
FA-36	0.92	104.06	18	270.26	6	3	4	65.54	2349.00	0.24	3.43	3.39
FA-37	0.18	130.08	18	253.21	7	5	4	62.70	3599.69	0.35	3.42	3.41
FA-38	0.09	118.81	16	224.22	6	6	3	60.15	1349.50	0.35	3.02	3.12
FA-39	0.39	113.01	16	225.20	6	5	3	57.41	3193.72	0.21	3.01	3.55
FA-40	0.84	92.78	16	241.27	5	4	3	62.99	2361.13	0.14	3.04	3.57
FA-41	0.68	124.29	18	254.19	7	4	4	59.95	3185.60	0.04	3.40	3.85
FA-42	0.59	113.01	16	225.20	6	5	3	57.41	3124.48	0.25	3.01	3.51
FA-43	0.89	107.22	16	226.18	6	4	3	54.66	2148.21	0.16	2.99	3.52
FA-44	1.34	86.99	16	242.25	5	3	3	60.25	1950.51	0.34	3.02	3.33
FA-46	0.84	92.78	16	241.27	5	4	3	62.99	2671.60	0.31	3.05	3.42
FA-47	1.13	86.99	16	242.25	5	3	3	60.25	1623.64	0.41	3.03	3.25
FA-49	0.92	141.36	20	282.20	8	4	5	65.25	3698.65	0.27	3.70	3.31
FA-50	0.83	130.08	18	253.21	7	5	4	62.70	4869.38	0.13	3.38	3.69
FA-51	1.13	124.29	18	254.19	7	4	4	59.95	3990.69	0.35	3.36	3.44
FA-53	0.41	130.08	18	253.21	7	5	4	62.70	3442.03	0.22	3.42	3.50
FA-54	0.32	118.81	16	224.22	6	6	3	60.15	1620.47	0.34	3.02	3.17
FA-55	0.62	113.01	16	225.20	6	5	3	57.41	2973.63	0.26	3.01	3.49

FA-58	0.39	113.01	16	225.20	6	5	3	57.41	2788.03	0.31	2.96	3.44
FA-59	0.68	107.22	16	226.18	6	4	3	54.66	2396.38	0.24	2.99	3.46
FA-62	1.06	92.78	16	241.27	5	4	3	62.99	1542.31	0.27	3.05	3.33
FA-63	1.36	86.99	16	242.25	5	3	3	60.25	1553.81	0.46	3.03	3.22
FA-65	0.77	104.06	17	238.20	6	3	4	58.29	4344.40	0.17	3.31	3.62
FA-66	0.68	92.78	15	209.20	5	4	3	55.74	4961.04	0.27	2.90	3.60
FA-67	0.98	86.99	15	210.19	5	3	3	52.99	4195.81	0.27	2.83	3.58
FA-69	-0.82	141.36	20	282.20	8	4	5	64.49	1688.65	0.38	3.47	2.97
FA-70	-0.67	130.08	18	253.21	7	5	4	61.04	2959.29	0.49	3.26	3.19
FA-71	0.69	124.29	18	254.19	7	4	4	59.48	1781.70	0.41	3.13	3.26
FA-72	0.25	104.06	18	270.26	6	3	4	65.84	1747.36	0.28	3.26	3.32
FA-73	-0.48	130.08	18	253.21	7	5	4	61.04	2226.10	0.33	3.30	3.21
FA-74	-0.33	118.81	16	224.22	6	6	3	57.59	3364.27	0.19	3.16	3.43
FA-75	1.03	113.01	16	225.20	6	5	3	56.03	2291.55	0.28	2.99	3.42
FA-76	0.58	92.78	16	241.27	5	4	3	62.39	1506.03	0.36	3.17	3.24
FA-77	-0.34	124.29	18	254.19	7	4	4	59.48	1566.95	0.39	3.28	3.22
FA-78	-0.18	113.01	16	225.20	6	5	3	56.03	1579.79	0.26	3.13	3.33
FA-79	1.17	107.22	16	226.18	6	4	3	54.47	2073.78	0.36	2.98	3.34
FA-80	0.73	86.99	16	242.25	5	3	3	60.83	1802.60	0.15	3.13	3.48
FA-81	0.05	104.06	18	270.26	6	3	4	65.84	1641.82	0.27	3.30	3.31
FA-82	0.20	92.78	16	241.27	5	4	3	62.39	2934.47	0.12	3.17	3.63
FA-83	1.56	86.99	16	242.25	5	3	3	60.83	5133.66	0.33	3.00	3.55
FA-84	1.11	66.76	16	258.32	4	2	3	67.19	1258.31	0.15	3.16	3.40
FA-85	-0.50	141.36	20	282.20	8	4	5	64.87	1309.65	0.39	3.63	2.89
FA-86	-0.59	130.08	18	253.21	7	5	4	62.32	2489.84	0.33	3.24	3.24
FA-87	-0.09	124.29	18	254.19	7	4	4	59.57	1218.06	0.28	3.22	3.25
FA-88	0.16	104.06	18	270.26	6	3	4	65.16	510.09	0.01	3.28	3.77
FA-89	0.15	141.36	20	282.20	8	4	5	64.87	2452.13	0.41	3.57	3.15
FA-90	-0.36	130.08	18	253.21	7	5	4	62.32	2090.73	0.33	3.22	3.33

FA-91	-0.29	124.29	18	254.19	7	4	4	59.57	1694.60	0.54	3.16	3.18
FA-92	0.39	104.06	18	270.26	6	3	4	65.16	1232.70	0.10	3.24	3.47
FA-93	0.00	104.06	17	238.20	6	3	4	57.91	1988.85	0.19	3.10	3.45
FA-94	-0.35	130.08	18	253.21	7	5	4	61.42	1778.62	0.49	3.42	3.18
FA-95	-0.44	118.81	16	224.22	6	6	3	58.87	2377.73	0.77	3.05	2.94
FA-96	0.07	113.01	16	225.20	6	5	3	56.12	1340.74	0.23	3.04	3.33
FA-98	0.30	130.08	18	253.21	7	5	4	61.42	2462.27	0.21	3.40	3.44
FA-99	-0.21	118.81	16	224.22	6	6	3	58.87	2551.25	0.36	3.05	3.25
FA-100	-0.14	113.01	16	225.20	6	5	3	56.12	1229.27	0.39	3.00	3.21
FA-101	0.54	92.78	16	241.27	5	4	3	61.71	558.39	0.45	3.07	2.99
FA-102	0.15	92.78	15	209.20	5	4	3	54.46	3063.91	0.20	2.94	3.56
FA-103	1.01	124.29	18	254.19	7	4	4	59.86	2566.85	0.46	3.34	3.28
FA-104	0.92	113.01	16	225.20	6	5	3	57.31	2624.82	0.27	2.97	3.46
FA-105	1.42	107.22	16	226.18	6	4	3	54.57	1630.44	0.36	2.96	3.29
FA-106	1.66	86.99	16	242.25	5	3	3	60.15	1014.35	0.01	2.99	3.96
FA-107	1.66	124.29	18	254.19	7	4	4	59.86	3704.29	0.22	3.29	3.53
FA-108	1.15	113.01	16	225.20	6	5	3	57.31	2381.79	0.10	2.94	3.66
FA-109	1.22	107.22	16	226.18	6	4	3	54.57	1587.16	0.23	2.89	3.39
FA-110	1.89	86.99	16	242.25	5	3	3	60.15	1292.67	0.11	2.95	3.50
FA-111	1.51	86.99	15	210.19	5	3	3	52.90	2167.98	0.15	2.79	3.57
FA-112	0.56	104.06	18	270.26	6	3	4	66.22	508.88	0.28	3.42	3.03
FA-113	0.47	92.78	16	241.27	5	4	3	63.67	988.86	0.36	3.06	3.17
FA-114	0.98	86.99	16	242.25	5	3	3	60.92	3122.98	0.27	3.04	3.48
FA-115	1.22	66.76	16	258.32	4	2	3	66.51	1461.59	0.01	3.10	4.03
FA-116	1.21	104.06	18	270.26	6	3	4	66.22	4240.07	0.26	3.40	3.51
FA-117	0.70	92.78	16	241.27	5	4	3	63.67	1996.09	0.19	3.05	3.46
FA-118	0.77	86.99	16	242.25	5	3	3	60.92	2113.27	0.06	3.00	3.73
FA-119	1.45	66.76	16	258.32	4	2	3	66.51	1550.35	0.17	3.07	3.43
FA-120	1.06	66.76	15	226.25	4	2	3	59.26	3912.04	0.20	2.94	3.61

FA-121	-0.25	141.36	20	282.20	8	4	5	64.87	2228.35	0.49	3.56	3.09
FA-122	-0.34	130.08	18	253.21	7	5	4	62.32	1655.03	0.52	3.18	3.18
FA-123	0.17	124.29	18	254.19	7	4	4	59.57	1872.80	0.28	3.09	3.36
FA-125	0.58	141.36	20	282.20	8	4	5	64.87	3916.41	0.36	3.52	3.28
FA-126	0.07	130.08	18	253.21	7	5	4	62.32	1813.57	0.47	3.16	3.23
FA-127	0.14	124.29	18	254.19	7	4	4	59.57	3017.85	0.29	3.11	3.45
FA-129	0.43	104.06	17	238.20	6	3	4	57.91	2030.14	0.13	2.98	3.56
FA-130	0.09	130.08	18	253.21	7	5	4	61.42	3957.12	0.28	3.40	3.48
FA-131	0.00	118.81	16	224.22	6	6	3	58.87	2643.05	0.26	3.04	3.33
FA-132	0.50	113.01	16	225.20	6	5	3	56.12	2740.56	0.23	3.03	3.49
FA-134	0.92	130.08	18	253.21	7	5	4	61.42	4520.59	0.32	3.37	3.48
FA-135	0.41	118.81	16	224.22	6	6	3	58.87	3348.67	0.21	3.01	3.43
FA-136	0.48	113.01	16	225.20	6	5	3	56.12	4228.28	0.21	2.86	3.63
FA-137	1.15	92.78	16	241.27	5	4	3	61.71	2165.76	0.18	3.04	3.49
FA-138	0.77	92.78	15	209.20	5	4	3	54.46	3022.97	0.05	2.74	3.89
FA-139	0.24	124.29	18	254.19	7	4	4	59.86	3721.68	0.28	3.40	3.47
FA-140	0.15	113.01	16	225.20	6	5	3	57.31	2574.85	0.24	3.04	3.47
FA-141	0.65	107.22	16	226.18	6	4	3	54.57	2163.28	0.17	3.02	3.51
FA-142	0.89	86.99	16	242.25	5	3	3	60.15	1780.92	0.01	3.08	4.07
FA-143	1.06	124.29	18	254.19	7	4	4	59.86	4175.83	0.28	3.37	3.49
FA-144	0.56	113.01	16	225.20	6	5	3	57.31	2283.22	0.30	3.01	3.40
FA-145	0.62	107.22	16	226.18	6	4	3	54.57	3310.89	0.19	2.96	3.58
FA-147	0.92	86.99	15	210.19	5	3	3	52.90	6133.62	0.37	2.86	3.59
FA-148	0.62	104.06	18	270.26	6	3	4	66.22	3014.87	0.34	3.40	3.38
FA-149	0.53	92.78	16	241.27	5	4	3	63.67	4122.82	0.39	3.04	3.46
FA-150	1.04	86.99	16	242.25	5	3	3	60.92	2156.74	0.39	3.03	3.33
FA-151	1.28	66.76	16	258.32	4	2	3	66.51	662.14	0.06	3.08	3.47
FA-152	1.45	104.06	18	270.26	6	3	4	66.22	2630.16	0.21	3.37	3.46
FA-153	0.94	92.78	16	241.27	5	4	3	63.67	1610.40	0.33	3.02	3.30

FA-154	1.01	86.99	16	242.25	5	3	3	60.92	1902.78	0.31	2.97	3.36
FA-155	1.69	66.76	16	258.32	4	2	3	66.51	887.05	0.04	3.04	3.63
FA-156	1.30	66.76	15	226.25	4	2	3	59.26	1492.20	0.12	2.86	3.53
FA-157	0.30	141.36	20	282.20	8	4	5	65.25	3254.06	0.24	3.69	3.31
FA-158	0.21	130.08	18	253.21	7	5	4	62.70	3835.71	0.28	3.38	3.47
FA-159	0.51	124.29	18	254.19	7	4	4	59.95	3404.81	0.29	3.36	3.44
FA-161	0.21	130.08	18	253.21	7	5	4	62.70	3591.72	0.22	3.42	3.51
FA-162	0.12	118.81	16	224.22	6	6	3	60.15	1787.02	0.49	3.02	3.11
FA-163	0.42	113.01	16	225.20	6	5	3	57.41	3262.29	0.25	3.01	3.51
FA-165	0.51	124.29	18	254.19	7	4	4	59.95	4618.86	0.23	3.37	3.56
FA-166	0.42	113.01	16	225.20	6	5	3	57.41	2936.62	0.13	2.96	3.64
FA-167	0.72	107.22	16	226.18	6	4	3	54.66	2543.00	0.31	3.00	3.42
FA-169	0.96	104.06	18	270.26	6	3	4	65.54	1911.54	0.30	3.43	3.30
FA-170	0.87	92.78	16	241.27	5	4	3	62.99	4026.98	0.28	3.04	3.53
FA-171	1.16	86.99	16	242.25	5	3	3	60.25	1726.80	0.63	3.02	3.17
FA-173	0.57	104.06	17	238.20	6	3	4	58.29	4471.90	0.13	3.22	3.70
FA-174	0.48	92.78	15	209.20	5	4	3	55.74	3391.37	0.44	2.78	3.43
FA-175	0.99	86.99	15	210.19	5	3	3	52.99	4040.74	0.03	2.76	4.06
FA-177	1.40	104.06	17	238.20	6	3	4	58.29	3837.04	0.14	3.18	3.65
FA-178	0.89	92.78	15	209.20	5	4	3	55.74	4841.39	0.36	2.80	3.55
FA-179	0.96	86.99	15	210.19	5	3	3	52.99	4863.77	0.21	2.70	3.69
FA-181	0.83	107.22	17	258.25	6	4	3	61.82	1454.07	0.29	3.22	3.28
FA-182	0.72	107.22	16	226.18	6	4	3	54.66	2543.00	0.31	3.00	3.42
FA-184	1.60	107.22	17	258.25	6	4	3	61.82	1675.77	0.11	3.15	3.53
FA-185	0.48	113.01	16	225.20	6	5	3	56.12	4228.28	0.21	2.86	3.63

Table S3. Elimination scores for the subset of ferulic acid derivatives chosen as the most promising, according to S^S.

	$S^{E,ADME2}$	$S^{E,ADME8}$	$S^{E,ADMET}$	$S^{E,ADMETSA}$
FA	1.33	4.12	9.28	11.13
FA-8	1.18	5.30	7.67	8.59
FA-12	0.56	4.01	6.99	8.03
FA-26	1.42	7.34	11.54	12.52
FA-41	0.97	6.21	10.11	10.80
FA-88	1.04	4.48	6.35	7.16
FA-106	0.65	4.09	5.50	6.56
FA-115	0.68	2.59	4.53	5.50
FA-118	1.05	4.50	7.04	8.11
FA-138	1.40	5.95	9.62	10.92
FA-142	1.00	4.44	6.76	7.74
FA-173	1.18	5.06	10.20	11.06
FA-175	1.29	5.17	10.12	11.40
Average	1.06	4.86	8.13	9.19

Table S4. Reference set of molecules, with some neuroprotective effects.

Compound (CAS)	Structure	Compound (CAS)	Structure
Acetylcarnitine (3040-38-8)	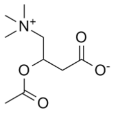	Masitinib (790299-79-5)	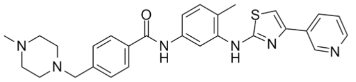
Amantadine (768-94-5)	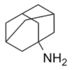	Melatonin (73-31-4)	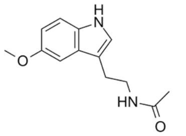
Apomorphine (58-00-4)	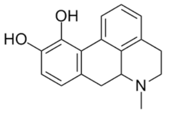	Memantine (19982-08-2)	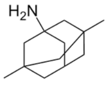
Baclofen (1134-47-0)	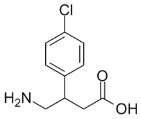	Modafinil (68693-11-8)	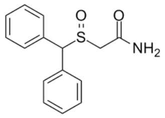
Benserazide (14919-77-8)	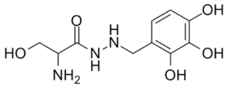	Piribedil (3605-01-4)	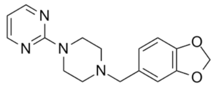
Benztropine (86-13-5)	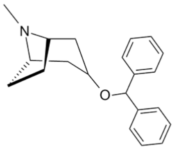	Pramipexole (104632-26-0)	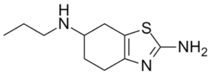
Biperiden (514-65-8)	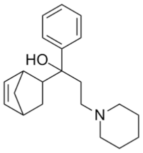	Procyclidine (77-37-2)	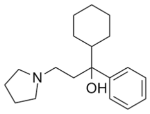
Bromocriptine (25614-03-3)	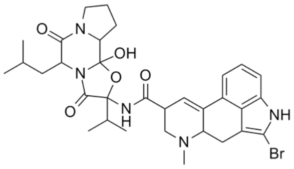	Remacemide (128298-28-2)	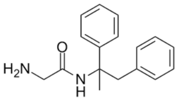
Cabergoline (81409-90-7)	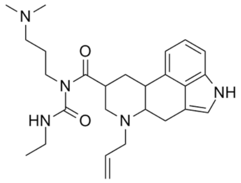	Riluzole (1744-22-5)	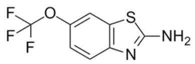
Carbidopa (28860-95-9)	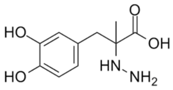	Rivastigmine (123441-03-2)	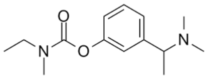

Curcumin<br>(458-37-7)	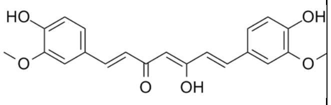	Ropinirole<br>(91374-21-9)	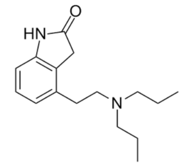
Dantrolene<br>(7261-97-4)	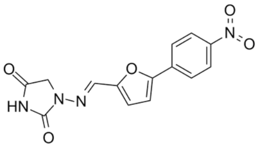	Selegiline<br>(14611-51-9)	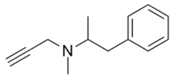
Donepezil<br>(120014-06-4)	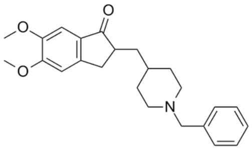	Tacrine<br>(321-64-2)	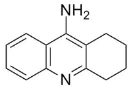
Entacapone<br>(130929-57-6)	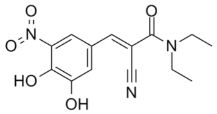	Tetrabenazine<br>(58-46-8)	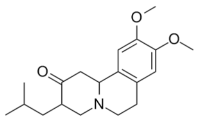
Galantamine<br>(357-70-0)	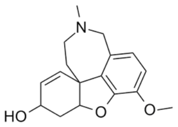	Tizanidine<br>(51322-75-9)	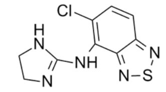
Ladostigil<br>(209349-27-4)	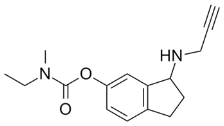	Tolcapone<br>(134308-13-7)	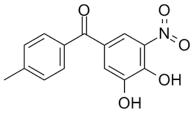
L-DOPA<br>(59-92-7)	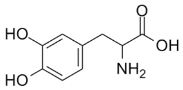	Trihexyphenid<br>yl<br>(144-11-6)	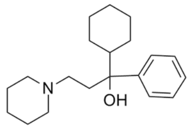
Lisuride<br>(18016-80-3)	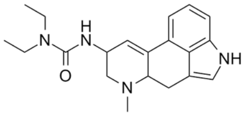		

Table S5. Pole strength (PS) values for the EPT approximation (P3) used to calculated ionization energies and electron affinities.

	PS (IE)	PS (EA)
**q= +1**		
FA-138	0.875	0.953
**q= 0**		
FA	0.879	0.913
FA-8	0.881	0.968
FA-26	0.879	0.970
FA-118	0.879	0.976
FA-138	0.877	0.968
FA-175	0.879	0.906
**q= -1**		
FA	0.881	0.973
FA-8	0.880	0.978
FA-26	0.883	0.980
FA-118	0.886	0.981
FA-138	0.883	0.982
FA-175	0.881	0.979
**q= -2**		
FA	0.876	0.983
FA-8	0.886	0.980
FA-26	0.877	0.983
FA-118	0.889	0.983
FA-138	0.878	0.984
FA-175	0.872	0.982
**q= -3**		
FA-118	0.875	0.985

Table S6. Equations concerning S^S construction.

$$S^S = S^{ADME} + S^T + S^{SA}$$

where

$$S^{ADME} = \frac{S^{logP} + S^{HB^D} + S^{HB^A} + S^{MW} + S^{^M R} + S^{^X A} + S^{RB} + S^{PSA}}{8}$$

$$S^T = \frac{S^{LD_{50}} + S^M}{2}$$

with

$$S^{logP} = \begin{cases} 1, & \text{if } -0.4 \le logP \le 5.0 \\ 0, & \text{otherwise} \end{cases} \qquad S^{HB^D} = \begin{cases} 1, & \text{if } HB^D \le 5 \\ 0, & \text{otherwise} \end{cases}$$

$$S^{HB^A} = \begin{cases} 1, & \text{if } HB^A \le 10 \\ 0, & \text{otherwise} \end{cases} \qquad S^{MW} = \begin{cases} 1, & \text{if } 160 \le MW \le 480 \\ 0, & \text{otherwise} \end{cases}$$

$$S^{^M R} = \begin{cases} 1, & \text{if } 40 \le {}^M R \le 130 \\ 0, & \text{otherwise} \end{cases} \qquad S^{^X A} = \begin{cases} 1, & \text{if } {}^X A \le 70 \\ 0, & \text{otherwise} \end{cases}$$

$$S^{RB} = \begin{cases} 1, & \text{if } RB \le 10 \\ 0, & \text{otherwise} \end{cases} \qquad S^{PSA} = \begin{cases} 1, & \text{if } PSA \le 140 \\ 0, & \text{otherwise} \end{cases}$$

$$S^{LD_{50}} = 1 + \log\left(\frac{{LD_{50}}^{dM}}{{LD_{50}}^{\overline{RefSet}}}\right) \qquad S^M = 1 + \log\left(\frac{M^{\overline{RefSet}}}{M^{dM}}\right) \qquad S^{SA} = 1 + \log\left(\frac{SA^{\overline{RefSet}}}{SA^{dM}}\right)$$

Table S7. Exclusion scores (S^E) equations.

$$S^{E,ADME2} = \left\lvert \frac{\log P_{\overline{RefSet}} - \log P_{dM}}{SD_{\log P}} \right\rvert + \left\lvert \frac{MW_{\overline{RefSet}} - MW_{dM}}{SD_{MW}} \right\rvert$$ $$S^{E,ADME8} = S^{E,ADME2} + \left\lvert \frac{PSA_{\overline{RefSet}} - PSA_{dM}}{SD_{PSA}} \right\rvert + \left\lvert \frac{{}^{X}A_{\overline{RefSet}} - {}^{X}A_{dM}}{SD_{{}^{X}A}} \right\rvert + \left\lvert \frac{HB^{A}{}_{\overline{RefSet}} - HB^{A}{}_{dM}}{SD_{HB^{A}}} \right\rvert + \left\lvert \frac{HB^{D}{}_{\overline{RefSet}} - HB^{D}{}_{dM}}{SD_{HB^{D}}} \right\rvert + \left\lvert \frac{RB_{\overline{RefSet}} - RB_{dM}}{SD_{RB}} \right\rvert + \left\lvert \frac{{}^{M}R_{\overline{RefSet}} - {}^{M}R_{dM}}{SD_{{}^{M}R}} \right\rvert$$ $$S^{E,ADMET} = S^{E,ADME8} + \left\lvert \frac{LD_{50\,\overline{RefSet}} - LD_{50\,dM}}{SD_{LD_{50}}} \right\rvert + \left\lvert \frac{M_{\overline{RefSet}} - M_{dM}}{SD_{M}} \right\rvert$$ $$S^{E,ADMETSA} = S^{E,ADMET} + \left\lvert \frac{SA_{\overline{RefSet}} - SA_{dM}}{SD_{SA}} \right\rvert$$

Table S8. Zero-point bond dissociation energies (BDE, in kcal/mol) for ferulic acid and its derivatives. Only the species with molar fractions $\geq 10^{-4}$ are included.

FA	**q=+1**	**q=0**	**q=-1**	**q=-2**	**q=-3**
Site a	---	98.09	97.94	96.96	---
Site b	---	85.15	82.48	---	---
FA-8	**q=+1**	**q=0**	**q=-1**	**q=-2**	**q=-3**
Site a	---	98.45	189.16	97.23	---
Site b	---	88.78	87.79	82.04	---
Site R5 (SH)	---	80.24	77.44	---	---
FA-26	**q=+1**	**q=0**	**q=-1**	**q=-2**	**q=-3**
Site a	---	98.93	97.79	96.98	---
Site b	---	85.12	81.89	71.30	---
Site R1 (OH)	---	83.06	77.29	---	---
FA-118	**q=+1**	**q=0**	**q=-1**	**q=-2**	**q=-3**
Site a	---	99.15	99.34	98.93	97.20
Site b	---	80.18	78.79	74.87	71.01
Site R3 (OH)	---	85.29	84.35	75.99	---
Site R4 (SH)	---	80.33	79.56	---	---
FA-138	**q=+1**	**q=0**	**q=-1**	**q=-2**	**q=-3**
Site a	100.02	98.90	98.62	97.02	---
Site b	89.30	83.97	74.88	---	---
FA-175	**q=+1**	**q=0**	**q=-1**	**q=-2**	**q=-3**
Site a	---	97.56	97.49	97.55	---
Site b	---	80.82	78.54	75.90	---
Site R2 (OH)	---	80.09	78.60	---	---

Table S9. Binding energies (ΔG_B, kcal/mol) for acid-base species of ferulic acid and its derivatives.

Compound	q= +1	q= 0	q= -1	q= -2	q= -3
COMT					
Ferulic acid	---	-5.49	-5.28	-5.31	---
FA-8	---	-5.03	-5.14	-4.93	---
FA-26	---	-4.98	-5.09	-4.8	---
FA-118	---	-5.46	-5.88	-6.09	-5.71
FA-138	-5.25	-5.1	-5.14	-5.2	---
FA-175	---	-5.35	-5.42	-5.49	---
MAO-B					
Ferulic acid	---	-7.32	-7.19	-6.98	---
FA-8	---	-6.88	-6.91	-6.75	---
FA-26	---	-7.37	-7.63	-7.31	---
FA-118	---	-6.35	-7.08	-7.24	-6.62
FA-138	-6.96	-7.02	-7.4	-7.1	---
FA-175	---	-6.96	-7.02	-6.92	---
AChE					
Ferulic acid	---	-7.25	-7.37	-7.51	---
FA-8	---	-6.45	-6.43	-6.42	---
FA-26	---	-7.93	-7.88	-8.08	---
FA-118	---	-6.82	-6.95	-6.94	-7.09
FA-138	-7.36	-7.39	-7.41	-7.67	---
FA-175	---	-6.81	-7.01	-7.02	---

Table S10. Complete set of weighted binding energies (ΔG^{w}_{B}, kcal/mol) for FA and its derivatives.

Compound	ΔG^{W}_{B} (Kcal/mol)			S_P
	COMT	MAO	AChE	
Ferulic acid	-5.28	-7.19	-7.37	3.78
FA-8	-5.14	-6.91	-6.43	3.50
FA-12	-4.97	-7.33	-4.38	3.09
FA-26	-5.09	-7.63	-7.88	3.93
FA-41	-5.23	-5.61	-6.59	3.34
FA-88	-4.84	-6.91	-3.79	2.87
FA-106	-5.25	-6.86	-4.44	3.08
FA-115	-4.67	-6.59	-4.30	2.90
FA-118	-5.90	-7.09	-6.95	3.79
FA-138	-5.12	-7.17	-7.40	3.76
FA-142	-5.31	-5.47	-5.83	3.17
FA-173	-5.23	-6.94	-7.35	3.73
FA-175	-5.42	-7.02	-7.01	3.70

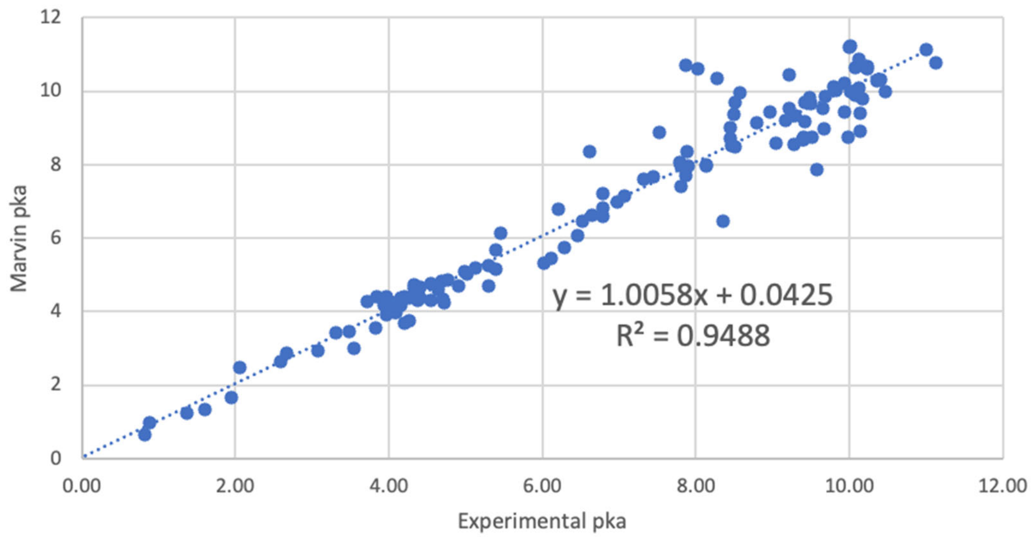

Figure S1. Correlation between pKa values estimated with Marvin software vs experimentally pKa values.

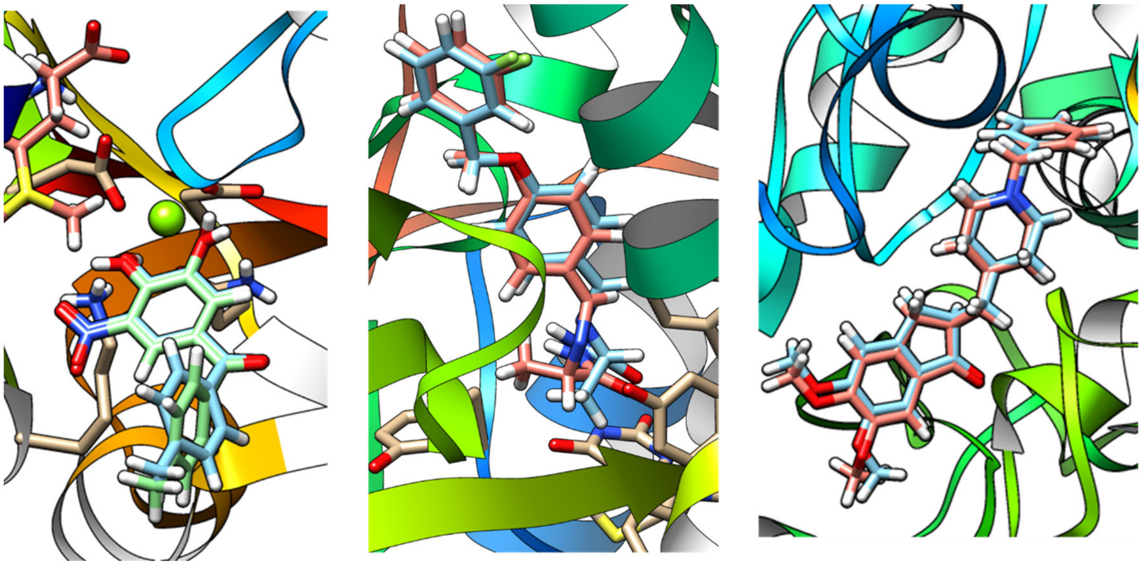

Figure S2. Redocking simulations of tolcapone in comt (left), Safrinamide in MAO-B (center) and Donopezil in AChE (right).

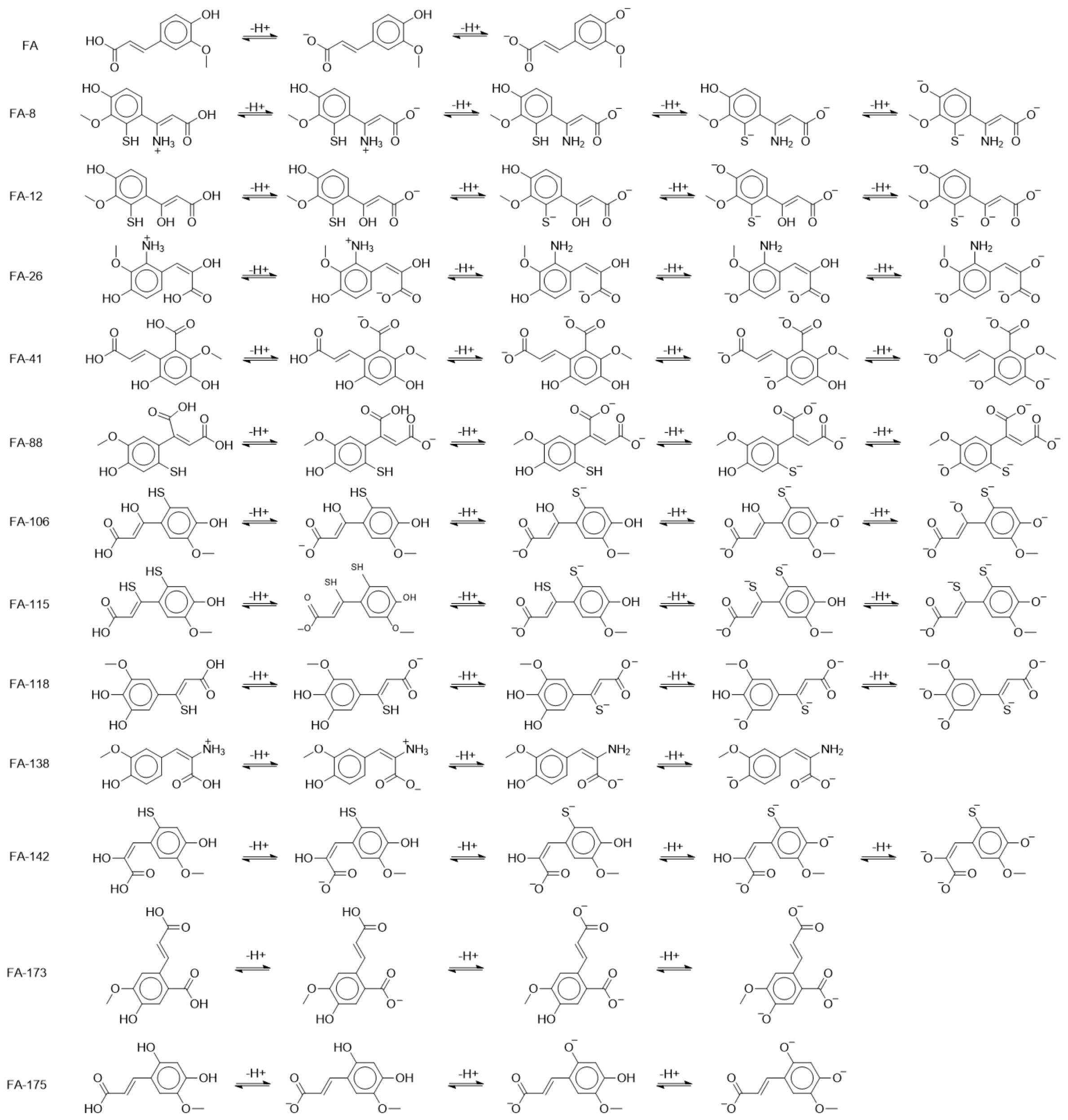

Figure S3. Deprotonation routes for the subset of ferulic acid derivatives chosen as the most promising, from their drug-like behavior.

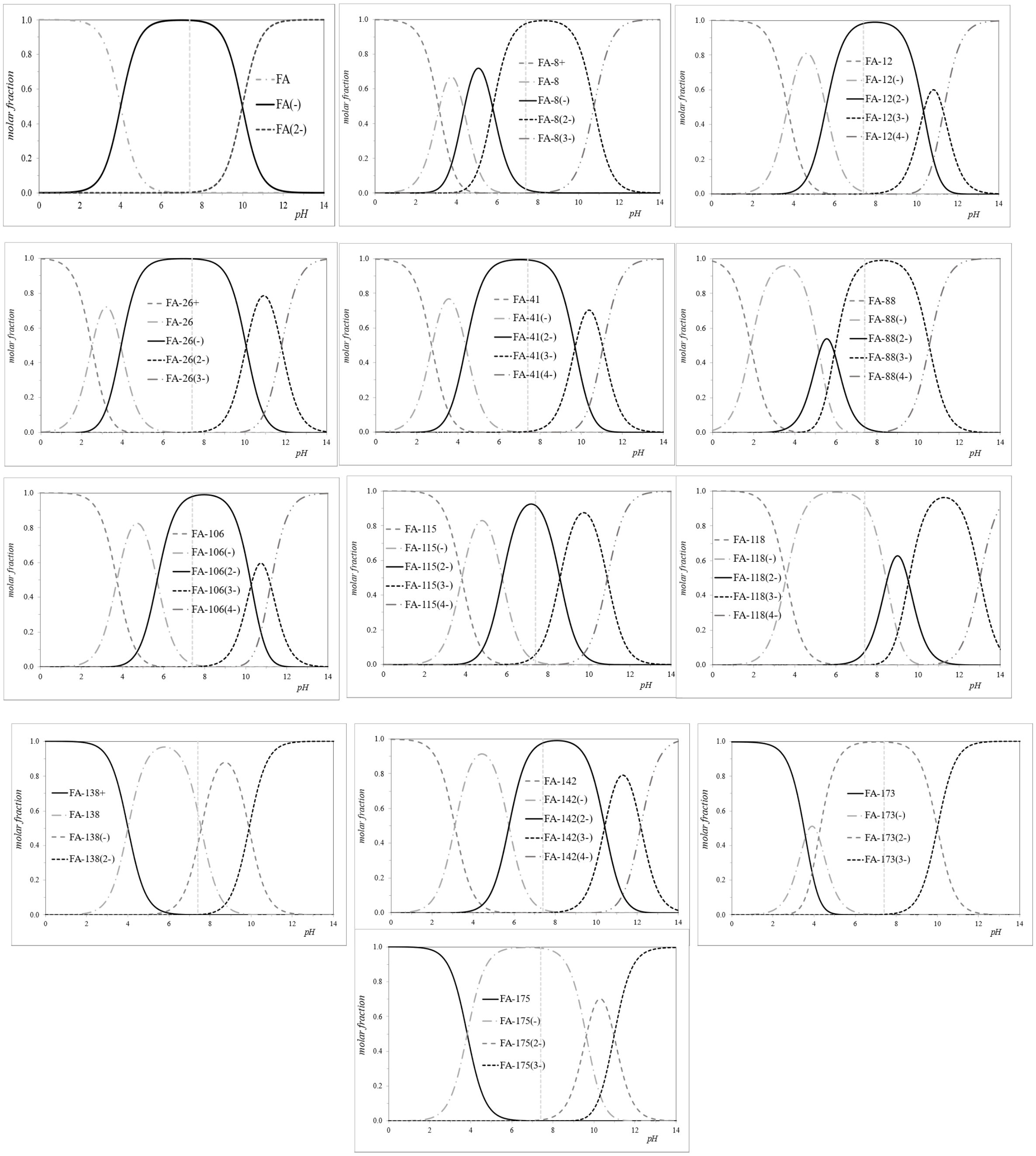

Figure S4. Distribution diagram of the acid-base species of ferulic acid derivatives. The vertical line landmarks the physiological pH (pH=7.4).
